# Local hydrology and seasonality outweigh the influence of *Phragmites australis* on coastal bacterial communities across two Baltic Sea bays

**DOI:** 10.1186/s40793-026-00979-4

**Published:** 2026-11-02

**Authors:** Fabio Ercoli, Helen Tammert, Veljo Kisand, Kätlin Blank, Carmen Kivistik, Daniel P. R. Herlemann

**Affiliations:** 1 Chair of Hydrobiology and Fisheries, Institute of Agricultural and Environmental Sciences, Estonian University of Life Sciences, Kreutzwaldi 5D, Tartu, 51006 Estonia; 2 Department of Biological and Environmental Science, University of Jyväskylä, P.O. Box 35, Jyväskylä, 40014 Finland; 3 Institute of Technology, University of Tartu, Tartu, Estonia; 4 Leibniz Institute for Baltic Sea Research Warnemünde, Seestrasse 15, 18119 Rostock, Germany

**Keywords:** Coastal microbial ecology, *Phragmites australis*, phyllosphere, Sediment, Seasonal dynamics, Salinity, Baltic Sea

## Abstract

**Background:**

Aquatic macrophytes shape microbial habitats in shallow coastal systems, yet the relative influence of plant-driven effects versus local hydrology and seasonal environmental variation remains poorly resolved. We investigated how *Phragmites australis* influences sediment, water, and phyllosphere bacterial communities in two shallow Baltic Sea bays, Haapsalu and Pärnu, that are approximately 83 km from each other.

**Results:**

Bacterial communities clustered strongly by substrate (sediment, water, and phyllosphere). Sediments exhibited higher 𝛼-diversity and stronger site specificity than water or phyllosphere habitats, with bulk sediments harboring greater richness than *Phragmites*-associated sediments. Seasonal 𝛼-diversity peaked in summer–autumn and reached minima in spring across sediments, whereas water communities showed weaker seasonality. β-diversity analyses revealed that sediments maintained lower seasonal turnover (i.e., more temporally stable) and site-specific communities compared to water and phyllosphere habitats, which showed rapid compositional shifts and higher seasonal dissimilarity. Pärnu Bay, which experiences stronger riverine influence, exhibits larger temporal shifts in both 𝛼- and β-diversity than Haapsalu Bay. Taxonomic differences between bays included the dominance of *Cyanobium* in Haapsalu Bay and *Synechococcus* in Pärnu Bay, most likely driven by site-specific hydrology and nutrient regimes. After accounting for environmental gradients, *P. australis* had only modest, context-dependent effects on the bacterial community composition.

**Conclusions:**

Local hydrology and seasonal variation, particularly riverine inputs and salinity dynamics, exert stronger control over coastal bacterial community composition in top-layer sediment and water than the presence of *P. australis*. While sediments act as relatively stable, site-specific reservoirs, water, and phyllosphere communities were more labile and seasonally dynamic. These findings underscore the importance of site-specific assessments in shallow coastal studies and caution against generalizing results across bays.

**Supplementary Information:**

The online version contains supplementary material available at https://doi.org/10.1186/s40793-026-00979-4.

## Introduction

Submerged and emergent macrophytes provide complex habitats that host diverse bacterial communities and strongly influence the surrounding microbial assemblages [11, 35]. Among the dominant shallow coastal macrophytes, *Phragmites australis* (common reed) is widespread across both coastal saline and inland freshwater wetlands [8, 33], playing a crucial role in shaping sediment microbial communities [5, 35]. As a perennial species with broad physiological tolerance, *P. australis* exhibits high salinity tolerance [13] and forms dense reed belts in brackish and freshwater wetlands, thereby driving local biodiversity and ecosystem functioning [8, 33, 50].

Aquatic macrophytes, such as *P. australis*, modify surrounding sediments through root oxygen release, nutrient uptake, exudation of organic compounds, and litter deposition, altering nutrient cycling as well as microbial activity and composition [15, 34]. However, *P. australis* undergoes distinct seasonal growth phases governed by temperature and nutrient availability [1]. During winter dormancy, aboveground biomass declines substantially, likely reducing belowground exudation and oxygen release. Consequently, root-associated bacterial communities may experience pronounced seasonal shifts. In reed-dominated sediments, plant-mediated changes in carbon to nitrogen (C:N) ratios and nutrient acquisition strategies can further structure microbial communities [2, 52]. While early-stage root-associated bacterial communities resemble adjacent bulk sediment and become increasingly plant-specific as plants grow and develop [6, 9], it remains unclear whether bulk sediment and root-associated bacteria communities follow similar or divergent seasonal patterns, particularly during winter dormancy.

Beyond plant effects, salinity is a major determinant of bacterial diversity and community composition in coastal wetlands [53, 31, 21]. In shallow estuaries and lagoons, salinity fluctuates markedly due to local hydrology, including river discharge, precipitation, and evaporation [30]. These fluctuations favor taxa with rapid physiological adaptability to changes in salinity. Similarly, coastal pH fluctuates more dynamically than in offshore water, driven by terrestrial organic inputs and phytoplankton dynamics. Intense photosynthesis reduces pCO_2_ and increases pH, whereas microbial respiration reduces the pH. Thus, coastal bacteria communities are exposed to dynamic environmental conditions that vary markedly across seasons due to fluctuation in salinity, temperature, and light availability [45]. Such seasonal dynamics are known to strongly influence both the structure and diversity of sediment and planktonic bacterial communities [16, 36].

Shallow semi-enclosed bays represent transitional zones between terrestrial and marine environments, characterized by strong environmental variability and different levels of anthropogenic activity. Along Baltic Sea shorelines, *P. australis* often dominates coastal areas, providing an ideal system to investigate plant and environmental effects on bacterial communities under fluctuating brackish conditions. In this study, we investigated the seasonal dynamics of bacterial community composition and diversity in four distinct niches: top-layer sediment (bulk sediment), phragmites-associated top-layer sediment (phragmites sediment), water, and submerged epiphytic biofilms (phyllosphere) over a 12-month period in two shallow brackish bays on the Estonian coast (Haapsalu Bay and Pärnu Bay). Pärnu Bay, which is larger (411 km²) and deeper (maximum depth 12 m), experiences strong freshwater discharge from the Pärnu River alongside urban and industrial harbor influences. In contrast, Haapsalu Bay (50 km² and maximum depth 5 m) is shallower, more sheltered, and receives only diffuse freshwater input.

Using 16S rRNA gene amplicon sequencing combined with generalized additive models (GAMs), this study provides the first comprehensive characterization of the bacterial community dynamics across these two bays in the Baltic Sea. Our specific objectives were to (1) assess whether bacterial community diversity differs between *Phragmites*-associated sediment and adjacent bulk sediment and between water and phyllosphere. (2.) Determine whether bacterial communities across sediments, water, and phyllosphere habitats exhibit distinct seasonal dynamics. We hypothesized that (i) *Phragmites*-associated sediments harbor bacterial communities that are compositionally distinct and more structured than adjacent bulk sediments due to plant-mediated selection. (ii) Sediments, water, and phyllosphere communities would display divergent seasonal patterns, reflecting habitat-specific environmental filtering and compositional turnover.

## Materials and methods

### Sampling

**Fig. 1:**
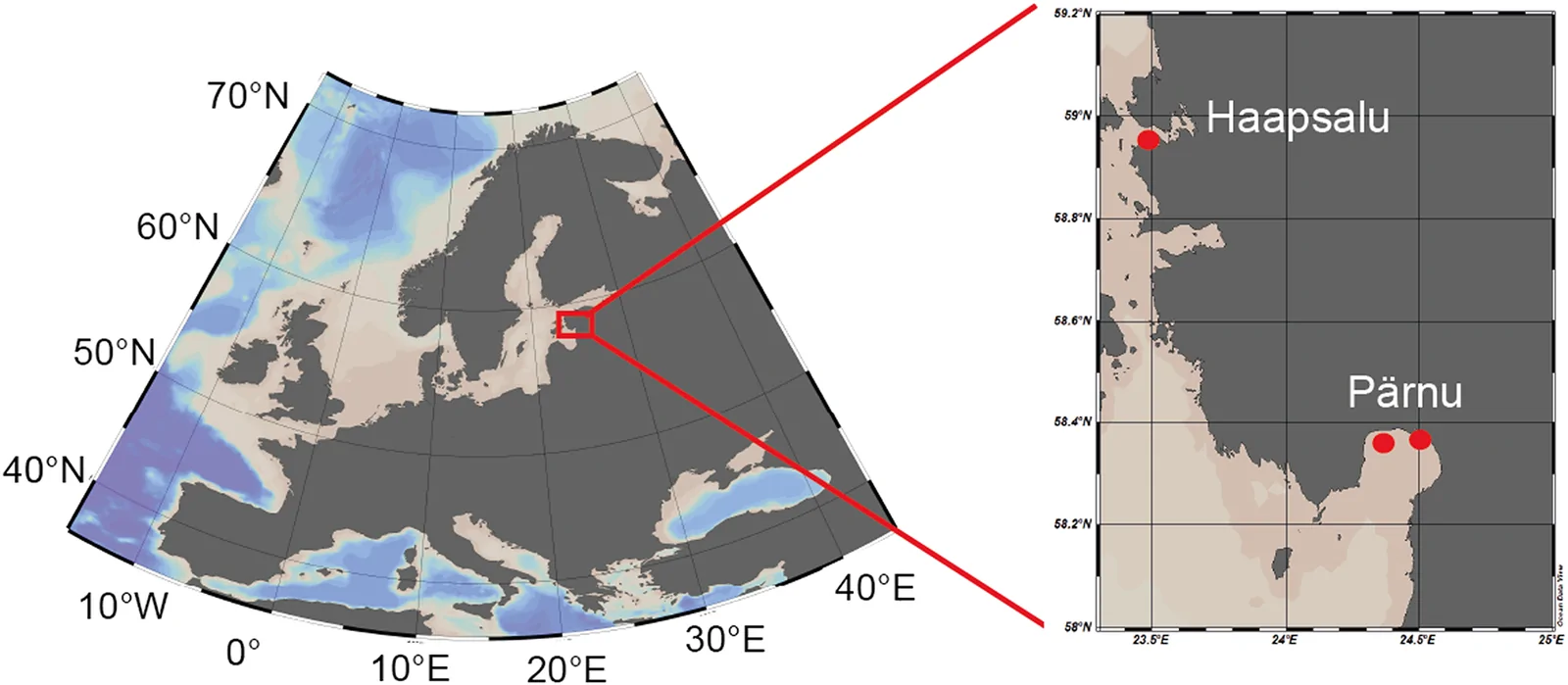
Map of bays where the samples were taken (red dots)

The sampling was conducted in two bays on the Estonian shore: (1) Pärnu Bay at the beaches “Keskrand” (58.37059° N, 24.50668° E) and “Valgerand” (58.38532° N, 24.35918° E) where only “Valgerand” has rich *P. australis* meadows in the water and (2) on the *P. australis* rich Haapsalu beach “Paralepa” (58.94305° N, 23.51696° E) (Fig. 1). The approximate distance between Pärnu Bay and Haapsalu Beach is 83 km. Sampling was performed at shallow sites at depths of 0.5–1 m.

Surface water and sediment samples were collected in triplicate at each of the three sites monthly from August 2020 to August 2021. Each replicate consisted of 1 L of water collected between the sediment and the surface by first rinsing 1 L sterile glass bottles three times before sampling water. Three replicates of sediment were collected from the top 2 cm in sterile 50 mL tubes at each sampling site and day. Sediment samples were taken within the *P. australis* reed belt phragmites (hereafter “phragmites sediment”) and 10 m far from the phragmites sediment (hereafter “bulk sediment”). The distance was used to evaluate the impact of *P. australis* on the sediment bacterial community and was chosen based on a previous sampling campaign [21].

Water and sediment samples were collected in January, February, and March by drilling through the surface ice layer and sampling below. No samples were taken in December 2020 because of unstable ice conditions. *Phragmites* epiphytic biofilm of submerged stems (hereafter phyllosphere) was sampled using sterile swabs and transported in sterile containers. The phyllosphere could not be sampled from January to March (except in January in Pärnu) because *Phragmites* was not visible under the ice cover. Temperature, pH, dissolved oxygen, and salinity were always measured on site using a YSI ProDSS multisensor.

Sea ice thickness in Haapsalu and Pärnu Bays was derived from satellite observations provided by the operational Baltic Sea - Sea Ice Concentration and Thickness Charts service [26] (https://doi.org/10.48670/moi-00132*).* All samples were transported back to the laboratory in darkened cool boxes within 3 h. In the laboratory, water was filtered through 0.22 μm filters using vacuum pumps. Filters, sediment, and swabs were kept frozen at -80 °C until the analysis.

### DNA extraction and sequence processing

DNA was extracted using the QIAGEN DNeasy PowerWater kit for water samples and the QIAGEN DNeasy PowerSoil Pro kit for bulk sediment and phragmites sediment samples, according to the manufacturer’s protocols. The swabs for phyllosphere samples were extracted using the DNeasy PowerSoil Pro kit, following the modifications described by Gebbe et al. [17]. The concentration of DNA was measured using a Qbit Fluorometer, and samples were amplified using primers Bakt_341F and Bakt_805R [20], which are universal primers for the V3-V4 region [27], normalized, and sequenced at an Illumina MiSeq platform at the SciLifeLab in Stockholm using the PE300 MiSeq Reagent kit ver 3. The resulting 2 × 300 bp paired end reads were processed using Trimmomatic (V0.36) [4] to remove Illumina-specific sequences and regions with low sequence quality (average quality score < Q20). The water samples from May did not pass the quality control. PCR primers were removed using the default values in Cutadapt (V2.3) [32]. The reads were paired (16 bp overlap, minimum length 300 bp) using the VSEARCH tool [41]. These were then taxonomically assigned using DADA2. Archaea, chloroplasts, and eukaryotes were removed because the primer does not provide sufficient coverage of these groups. Samples with fewer than 950 reads were removed for further analysis based on rarefaction analysis (Supplementary Fig. 1).

### Statistical analysis

After quality control, a total of 4,436,792 high-quality sequences of 333 samples were obtained. Of the bulk sediment, Pärnu Bay contained 66 samples and Haapsalu Bay 32 samples. For phragmites sediment, Pärnu Bay contained 32 samples and Haapsalu Bay 30 samples. The water in Pärnu Bay was represented by 57 samples and Haapsalu Bay by 26 samples. For the phyllosphere, Pärnu Bay contained 48 samples and Haapsalu Bay 42 samples.

To normalize for variation in the read yield between samples (950-46785 reads) and account for the compositionality of the data, centered log ratio (clr) transformation was applied to evaluate the bacterial community composition (β-diversity). This compositional data analysis approach [18] separates data variance. The data were split into sub-datasets where each substrate (bulk sediment, phragmites sediment, water, and phyllosphere) was tested separately to assess the temporal effects and impact of the environmental factors between sampling sites. To address the impact of *P. australis*, the subset of data composed of samples from each substrate (phragmites sediment vs. bulk sediment, and water vs. phyllosphere) was tested for the different seasons described in this region of the Baltic Sea [39]. Distance matrices were produced in PAST based on the CLR transformed data using Euclidean distance (Aitchison distance). Similarly, the principal component analysis (PCA) was performed on the Aitchison distance. To identify bacterial taxa associated with specific habitats (bulk sediment, phragmites sediment, phyllosphere, and water) and bays (Haapsalu and Pärnu), indicator species analysis was performed using the multipatt function in the R package indicspecies [7]. Analyses were conducted on dominant taxa reaching > 8% relative abundance, consistent with the taxa selected for the heatmaps. Indicator species analyses were performed to identify taxa significantly associated with bulk sediment vs. phragmites sediment and water vs. phyllosphere within each bay and between Haapsalu and Pärnu within each substrate. Statistical significance was assessed using 9999 permutations (p-value < 0.05). To assess associations between environmental conditions and temporal variation in dominant bacterial assemblages, relative abundances of the dominant taxa were centered log-ratio (CLR) transformed and subjected to principal component analysis (PCA). Temperature, salinity, dissolved oxygen (DO), and pH were fitted onto the resulting ordinations using the envfit function in the R package vegan with 9999 permutations. Environmental variables with p-value < 0.05 were considered significantly associated with variation in community composition.

Richness and diversity indexes were calculated after subsampling and bootstrapping to the lowest number of reads per sample (950 reads), using Explicet [40]. Diversity indices and environmental features were tested for normality before statistical analyses using the Shapiro-Wilk test and log-transformed if normality assumptions were not met. Environmental variables (salinity, temperature, pH, and dissolved oxygen (DO) were tested for differences between sites using a linear mixed effects model (lme4 package) [3], including sites as fixed effects and months as a random effect. In addition to the parameters measured during the field campaign, data from the Estonian Marine Institute collected during a monitoring program for Pärnu Bay and Haapsalu Bay were included. This included Chlorophyll-a (Chl-a), total nitrogen (N_tot_), and total phosphorus (P_tot_) of Haapsalu and Pärnu Bays from April to October 2021. If significant effects were found (p-value < 0.05), pairwise comparisons with the Tukey post hoc test were performed to determine which specific pairs of groups differ significantly from each other. Also, to provide additional information on riverine organic matter input to Pärnu Bay, total organic carbon (TOC) concentrations measured by the Estonian Environmental center in 2020 in the Pärnu River in proximity to the bay (58.3914° N, 24.4897° E) were included for four seasonal sampling periods: February, April, August, and October. Seasonal and spatial differences in 𝛼- and β-diversity indexes across substrates (water, bulk sediment, phragmites sediment, and phyllosphere) and sites were tested using generalized additive models (GAM) with a Gaussian error distribution and smoothness parameters estimated by restricted maximum likelihood (REM). Chao1 and Shannon indexes were used as measures of 𝛼-diversity, while β-diversity was represented by community dissimilarity. Temporal variation was modeled as a smooth function of sampling month using cyclic cubic regression splines, with separate smooths fitted for each substrate or site. Substrate and site were additionally included as parametric terms to account for differences in their overall levels, and salinity was included as a covariate in between-site model. Seasonal patterns were visualized as partial effects of the fitted smooth terms using the gratia package [44]. The plotted smooths represent the estimated contribution of the seasonal smooth to the model linear predictor after accounting for the other terms in the model. The smooth terms were centered around zero, allowing the model to distinguish the overall level of the response and its seasonal variation. Therefore, the partial effect plots represent seasonal deviations from the fitted overall level after accounting for the other terms in the model. Consequently, for α-diversity, positive partial effects indicate months in which the seasonal component predicts higher Chao1 or Shannon diversity relative to its centered reference, whereas negative values indicate lower diversity. For β-diversity, positive partial effects indicate a seasonal contribution toward greater community dissimilarity, whereas negative values indicate a contribution toward greater community similarity. Differences in temporal patterns between substrates within a site and between sites for the same substrate were evaluated by calculating pairwise differences between the corresponding fitted smooths [43]. The difference smooth represents the estimated difference between the two seasonal smooth functions at each time point. A temporal difference was considered statistically significant during periods in which the 95% confidence interval of the difference smooth did not include zero. Thus, significance refers to specific periods in which the estimated seasonal trajectories differed, rather than to a difference inferred solely from visual separation of the individual smooth curves. The GAM visualization and smooth comparisons were run using the gratia package in R [44]. All analyses were performed in R version 4.4.1 [37].

## Result

### Physicochemical features and differences between sites

Based on the seasonal categorization, the sampling campaign comprised five winter (December-February) samples, nine spring (March-May) samples, twenty-two summer (June-August) samples, and six autumn (September-November) samples. Ice cover was present in both bays from January 2021 to April 2021. However, in Pärnu Bay, it persisted longer (20 days) and was thicker (≈ 10 cm) than in Haapsalu Bay. Water temperature, salinity, dissolved oxygen (DO), and pH were evaluated across sampling locations (Paralepa, Keskrand, and Valgerand) over the study period. None of these environmental parameters differed significantly between Keskrand and Valgerand in Pärnu (*p* > 0.05). Consequently, due to their environmental and geospatial similarity, Keskrand and Valgerand were grouped into the site representing Pärnu Bay, whereas Paralepa represented Haapsalu Bay (Fig. 1).

**Fig. 2:**
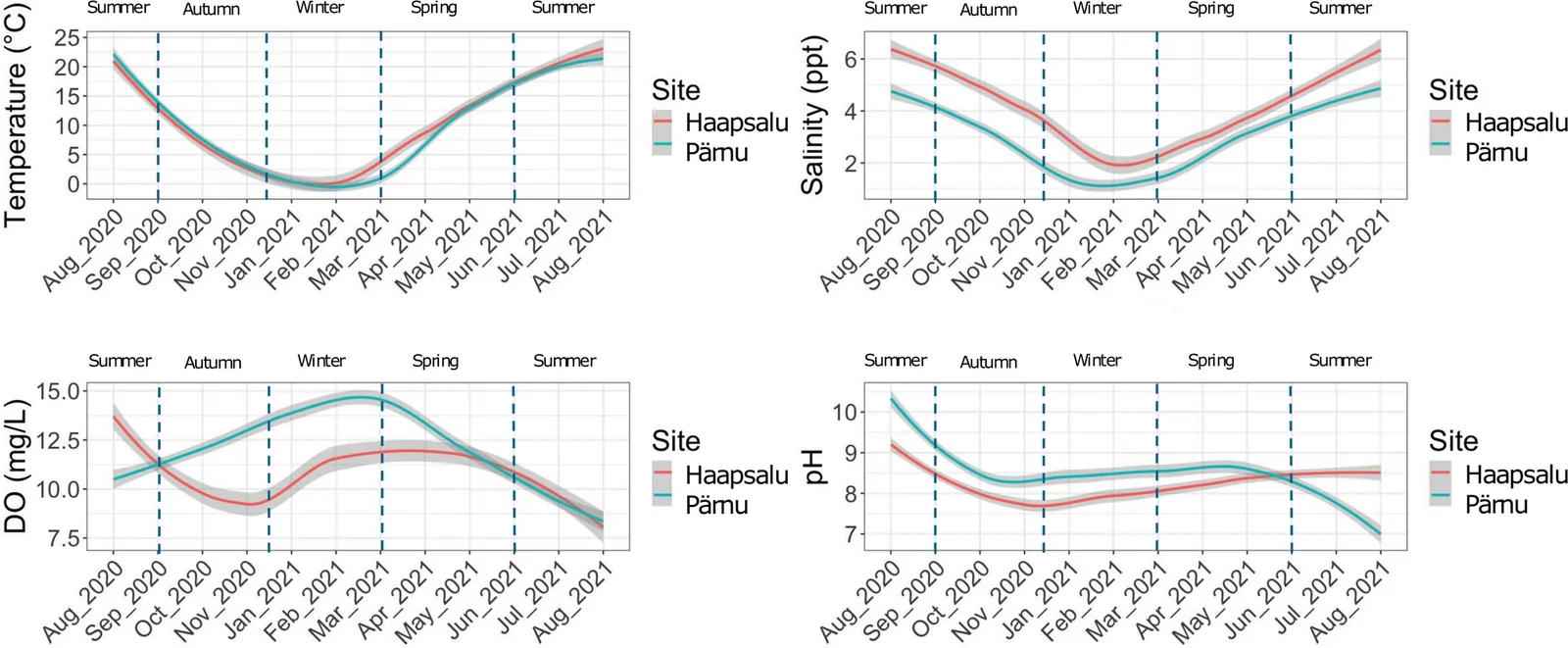
Seasonal variation of environmental variables in Haapsalu and Pärnu Bays is represented by smooth lines with a 95% confidence interval (gray shadow)

As expected, strong seasonal variation in environmental variables was observed across the months (Fig. 2). Water temperature was lowest in winter, ranging from  -0.6 to -1 °C, and highest in summer (9.2–25.8 °C), while spring and autumn temperature was between  -0.6 and 15.1 °C, and 3.9–8.5 °C. Salinity ranged from winter minimum of 0.4 ppt to summer maximum of 6.7 ppt. The lowest and highest dissolved oxygen (6.9–22.6 mg/L) and pH (6.1–11.5) were found in summer.

After accounting for the monthly variation, Pärnu Bay had significantly lower temperature (p-value < 0.001) and salinity (p-value < 0.001) but significantly higher pH (p-value < 0.001) and DO (p-value < 0.001) compared to Haapsalu Bay. The strongest differences occurred in temperature between February 2021 and July 2021, and for salinity between August 2020 and August 2021, for dissolved oxygen (DO) between September 2020 and April 2021, and for pH between August 2020 and May 2021 (Fig. 2). Although Chl-a (p-value = 0.05), N_tot_ (p-value = 0.54) and P_tot_ (p-value = 0.06) did not differ significantly between the two bays from April to October 2021, there is an indication of higher Chl-a in Pärnu Bay (Supplementary Fig. 2). Pärnu River TOC concentrations varied seasonally (12–23 mgC/L), with the highest concentration in winter (February, 23 mgC/L) and summer (August, 17 mgC/L) and the lowest in autumn (October, 12 mgC/L).

### Seasonal variation in α-diversity between substrates

The bacterial 𝛼-diversity was estimated based on the Chao1 and Shannon indexes. Average Chao1 was higher in the bulk sediment (12–191 ASVs) and phragmites sediment (13–200 ASVs) than in the phyllosphere (8-150 ASVs) and water (6-150 ASVs).

**Fig. 3:**
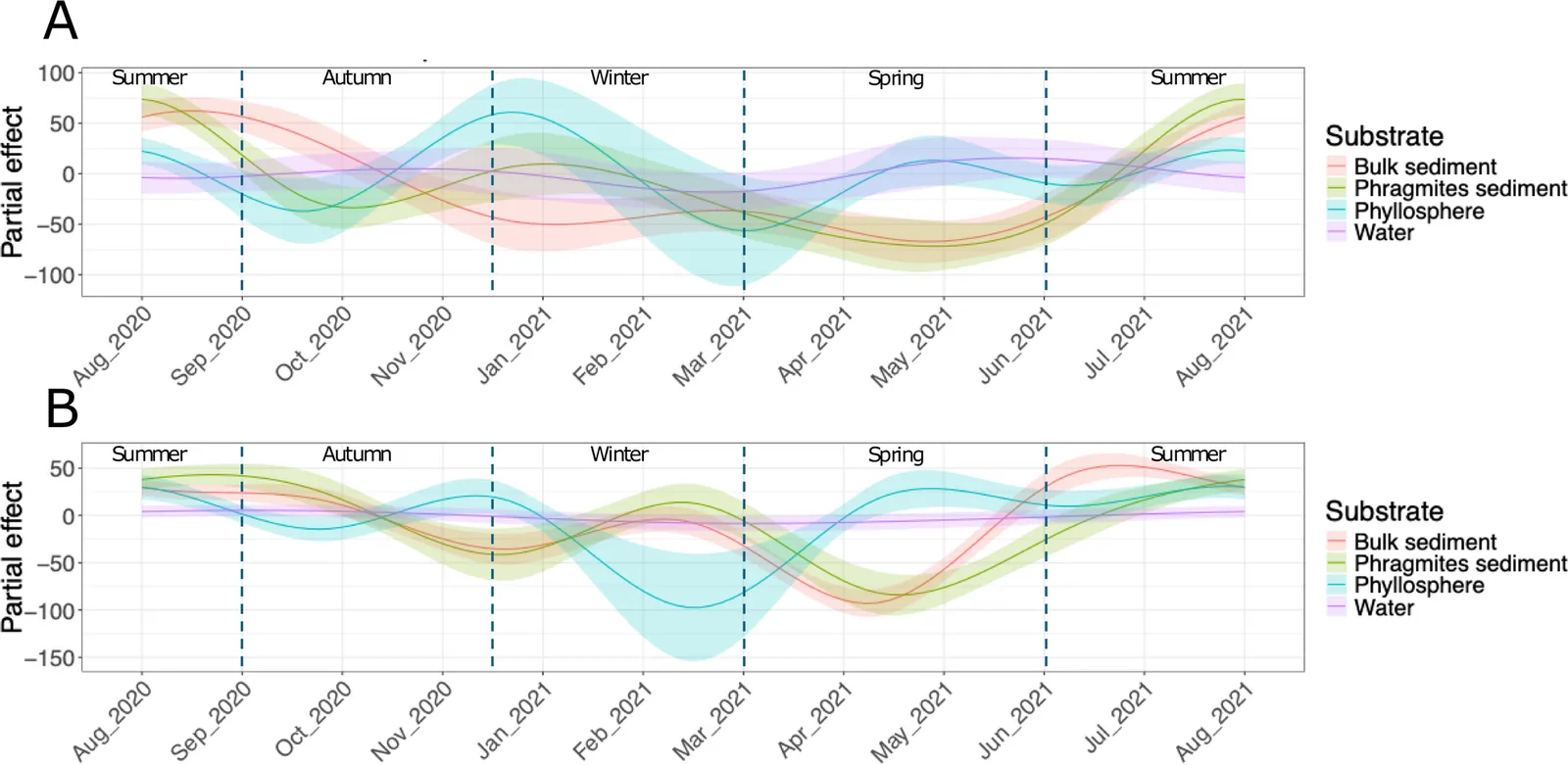
Chao1 seasonality in Haapsalu (**A**) and Pärnu (**B**) Bays, per substrate, represented by smooth lines and their associated 95% confidence intervals (shadow area) calculated by GAMs. Smooth terms are centered at zero, and positive and negative values indicate higher and lower 𝛼-diversity. Significant differences between paired temporal smooths were identified from difference smooths when their 95% confidence intervals excluded zero

**Fig. 4:**
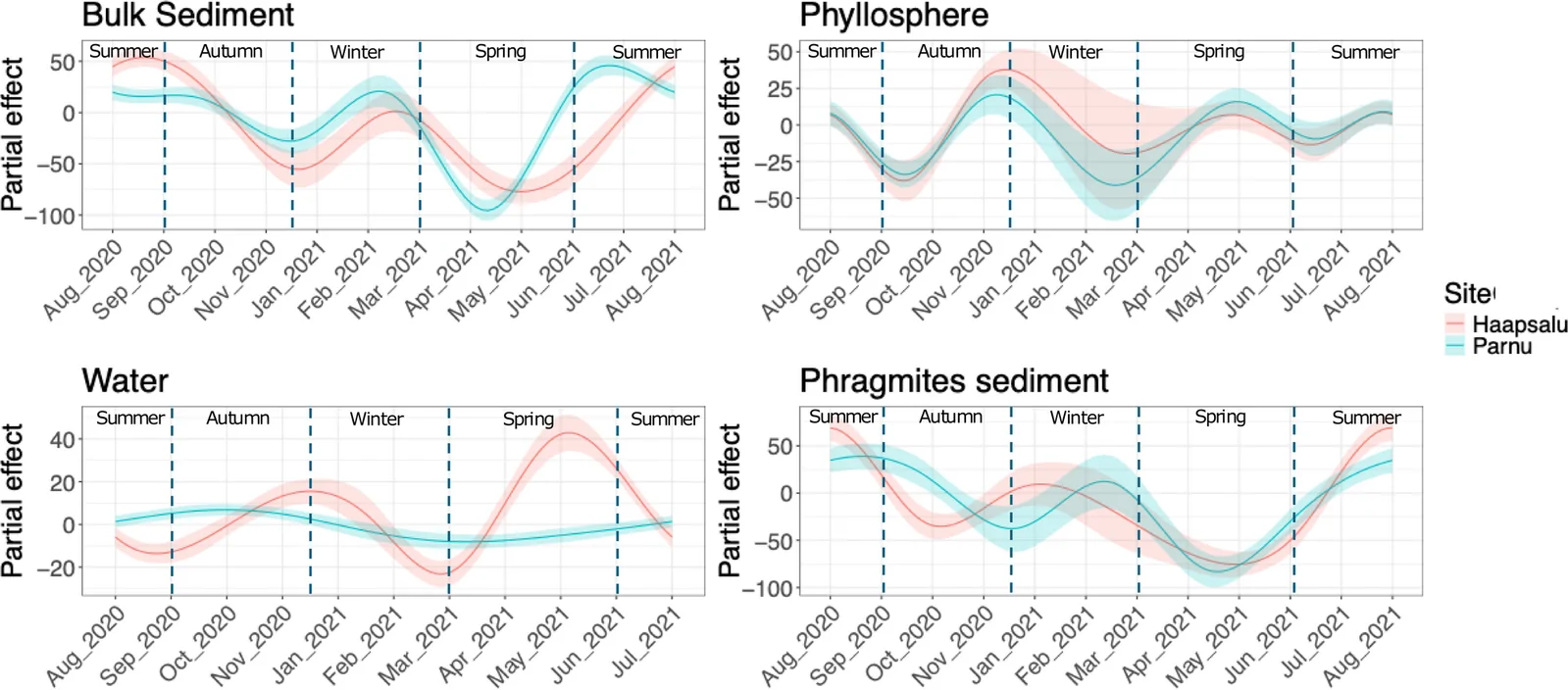
Chao1 seasonality in each substrate in Haapsalu and Pärnu Bays is represented by smooths, with their associated 95% confidence intervals (shaded areas) calculated by GAMs. Smooth terms are centered at zero; therefore, positive and negative values indicate higher and lower 𝛼-diversity. Significant differences between paired temporal smooths were identified from difference smooths when their 95% confidence intervals excluded zero

Besides peaks in November and June, February and March were the lowest Chao1 and Shannon, found in the water, especially during ice cover (Supplementary Figs. 3 and 4). The bulk sediment and phragmites sediment had lower 𝛼-diversity from March to June than water and the phyllosphere (Supplementary Figs. 2 and 3).GAM results showed that both Chao1 (Haapsalu Bay: adj R^2^ = 0.66; deviance explained = 71%; Pärnu Bay: adj R^2^  = 0.74; deviance explained = 76.5%) and Shannon (Haapsalu Bay: adj R^2^  = 0.64; deviance explained = 69.5%; Pärnu Bay: adj R^2^  = 0.78; deviance explained = 80.3%) indexes have significant seasonal variation in the bulk sediment (p-value < 0.001; p-value < 0.001), phyllosphere (p-value < 0.001; p-value < 0.001), and phragmites sediment (p-value < 0.001; p-value < 0.001) in Haapsalu and Pärnu Bays (Fig. 3; Supplementary Fig. 5). While the seasonal variation of Chao1 in water was significant in Pärnu and not in Haapsalu Bay (p-value < 0.001; p-value = 0.19), the seasonal variation of the Shannon Index in water was significant in both bays (p-value < 0.001) (Supplementary Fig. 5). Seasonal variation differences in 𝛼-diversity indexes (Chao1 and Shannon) were examined by comparing paired smooths between different substrates in each bay. Generally, smooth comparison models revealed that bulk sediment, phragmites sediment, water and phyllosphere 𝛼-diversity followed different temporal patterns (Supplementary Figs. 6, 7, 8 and 9). However, no differences in seasonal variation were found between bulk sediment and phragmites sediment, except during autumn and winter in Haapsalu Bay (Figs. 3 and 4; Supplementary Figs. 6 and 7), and during summer in Pärnu Bay (Figs. 3 and 4; Supplementary Figs. 8 and 9). Seasonal difference patterns were found between the phyllosphere and water in Haapsalu and Pärnu Bays during winter (Figs. 3 and 4; Supplementary Figs. 6, 7, 8 and 9), and in Pärnu Bay in spring and summer (Figs. 3 and 4; Supplementary Figs. 8 and 9).

### Seasonal variation of α-diversity in substrates between bays

GAMs were also used to explore differences in Chao1 and Shannon indexes and seasonal variation of each substrate between Haapsalu and Pärnu Bays. Bulk sediment seasonal variation between Pärnu and Haapsalu Bays was indicated for Chao1 and Shannon during spring and summer (Fig. 4; Supplementary Figs. 10, 11 and 12), while phragmites sediment differed in autumn. The phyllosphere did not show significant differences in seasonal smooths between the two bays, while the water showed significant differences in spring and summer (Fig. 4; Supplementary Figs. 9, 10, and 11). This suggests that seasonal differences in 𝛼-diversity exist between the bays in water and bulk sediments, particularly in summer, and in phragmites sediment in autumn.

### Substrate effects on α-diversity

The presence of reed in Haapsalu Bay had no significant effects on Chao1 and Shannon bulk sediment vs. phragmites sediment (p-value = 0.67; p-value = 0.62), or water vs. phyllosphere (p-value = 0.08; p-value = 0.36) (Table 1). In Pärnu Bay, the Chao1 index between bulk sediment and phragmites sediment was somewhat significant (p-value = 0.03), whereas the Shannon index was not significant (p-value = 0.12) (Table 1). The only weakly significant difference between water and phyllosphere was detected for Shannon index in Pärnu Bay (p-value = 0.04) but not for Chao1 (p-value = 0.19) (Table 1).

**Table 1:** GAM results of fixed effect factor (substrates) on Chao1 and Shannon indexes within each site (Haapsalu and Pärnu)

Site	Diversity index	Substrate reference	Substrate comparison	Estimate	Std. Error	t value	*p*-value
Haapsalu	Chao1	Bulk sediment	Phragmites sediment	-2.983	7.148	-0.417	0.677
Haapsalu	Chao1	Bulk sediment	Phyllosphere	-31.875	7.972	-3.998	**< 0.001**
Haapsalu	Chao1	Bulk sediment	Water	-46.476	7.591	-6.122	**< 0.001**
Haapsalu	Chao1	Phragmites sediment	Phyllosphere	-28.891	8.064	-3.582	**< 0.001**
Haapsalu	Chao1	Phragmites sediment	Water	-43.493	7.687	-5.657	**< 0.001**
Haapsalu	Chao1	Phyllosphere	Water	-14.601	8.459	-1.726	0.08
Haapsalu	Shannon	Water	Phyllosphere	0.162	0.178	0.911	0.364
Haapsalu	Shannon	Water	Phragmites sediment	0.612	0.162	3.761	**< 0.001**
Haapsalu	Shannon	Water	Bulk sediment	0.684	0.160	4.256	**< 0.001**
Haapsalu	Shannon	Phyllosphere	Phragmites sediment	0.449	0.164	2.731	**0.007**
Haapsalu	Shannon	Phyllosphere	Bulk sediment	0.521	0.162	3.208	**0.001**
Haapsalu	Shannon	Phragmites sediment	Bulk sediment	0.072	0.145	0.497	0.619
Pärnu	Chao1	Bulk sediment	Phragmites sediment	-10.986	5.032	-2.182	**0.03**
Pärnu	Chao1	Bulk sediment	Phyllosphere	-51.351	5.621	-9.134	**< 0.001**
Pärnu	Chao1	Bulk sediment	Water	-58.874	4.107	14.333	**< 0.001**
Pärnu	Chao1	Phragmites sediment	Phyllosphere	-40.364	6.437	-6.270	**< 0.001**
Pärnu	Chao1	Phragmites sediment	Water	-47.887	5.167	-9.266	**< 0.001**
Pärnu	Chao1	Phyllosphere	Water	-7.523	5.742	-1.309	0.191
Pärnu	Shannon	Water	Phyllosphere	0.278	0.132	2.106	**0.04**
Pärnu	Shannon	Water	Phragmites sediment	1.280	0.118	10.831	**< 0.001**
Pärnu	Shannon	Water	Bulk sediment	1.455	0.094	15.425	**< 0.001**
Pärnu	Shannon	Phyllosphere	Phragmites sediment	-1.001	0.147	-6.813	**< 0.001**
Pärnu	Shannon	Phyllosphere	Bulk sediment	-1.176	0.128	-9.145	**< 0.001**
Pärnu	Shannon	Phragmites sediment	Bulk sediment	-0.174	0.114	-1.52	0.128

### Difference between bays on α-diversity

In general, the site effect from GAM results indicated that Chao1 and Shannon indexes in the phyllosphere (p-value = 0.38; p-value = 0.84), bulk sediment (p-value = 0.08; p-value = 0.03), and phragmites sediment (p-value = 0.92; p-value = 0.49) did not differ significantly between Haapsalu and Pärnu Bays, except for the Shannon index in the phyllosphere (Table 2). There is no overall difference between the bays in all substrates. However, the 𝛼-diversity (Shannon) in water differed significantly between Haapsalu and Pärnu (p-value = 0.002) (Table 2). Salinity significantly affected the Chao1 index (p-value = 0.04) in bulk sediment between Haapsalu and Pärnu Bays.

**Table 2:** GAM results of fixed effect factor (sites) on Chao1 and Shannon indexes in each substrate between the two sites (Haapsalu and Pärnu)

Diversity index	Site comparison	Substrate	Estimate	Std. Error	t value	*p*-value
Chao1	Haapsalu-Pärnu	Bulk sediment	13.507	7.876	1.715	0.08
Chao1	Haapsalu-Pärnu	Phragmites sediment	-1.096	12.009	-0.091	0.92
Chao1	Haapsalu-Pärnu	Phyllosphere	8.281	9.481	0.873	0.38
Chao1	Haapsalu-Pärnu	Water	-16.124	5.106	-3.158	0.06
Shannon	Haapsalu-Pärnu	Bulk sediment	0.314	0.145	2.163	**0.03**
Shannon	Haapsalu-Pärnu	Phragmites sediment	0.170	0.245	0.696	0.49
Shannon	Haapsalu-Pärnu	Phyllosphere	-0.0415	0.209	-0.198	0.84
Shannon	Haapsalu-Pärnu	Water	-0.418	0.135	-3.085	**0.002**

### β-diversity seasonal variation

GAMs were used to evaluate the seasonal turnover and similarity of community composition (β-diversity) between paired substrates in Haapsalu and Pärnu Bays and in each substrate between the two bays. In this method, higher distance (partial effect > 0) values indicate greater dissimilarity (more distinct communities), whereas lower distance values (partial effect < 0) indicate more similar communities. Salinity differed significantly between Haapsalu and Pärnu Bays and was included in the models to account for its effects on β-diversity seasonality between Haapsalu and Pärnu Bays.

**Fig. 5:**
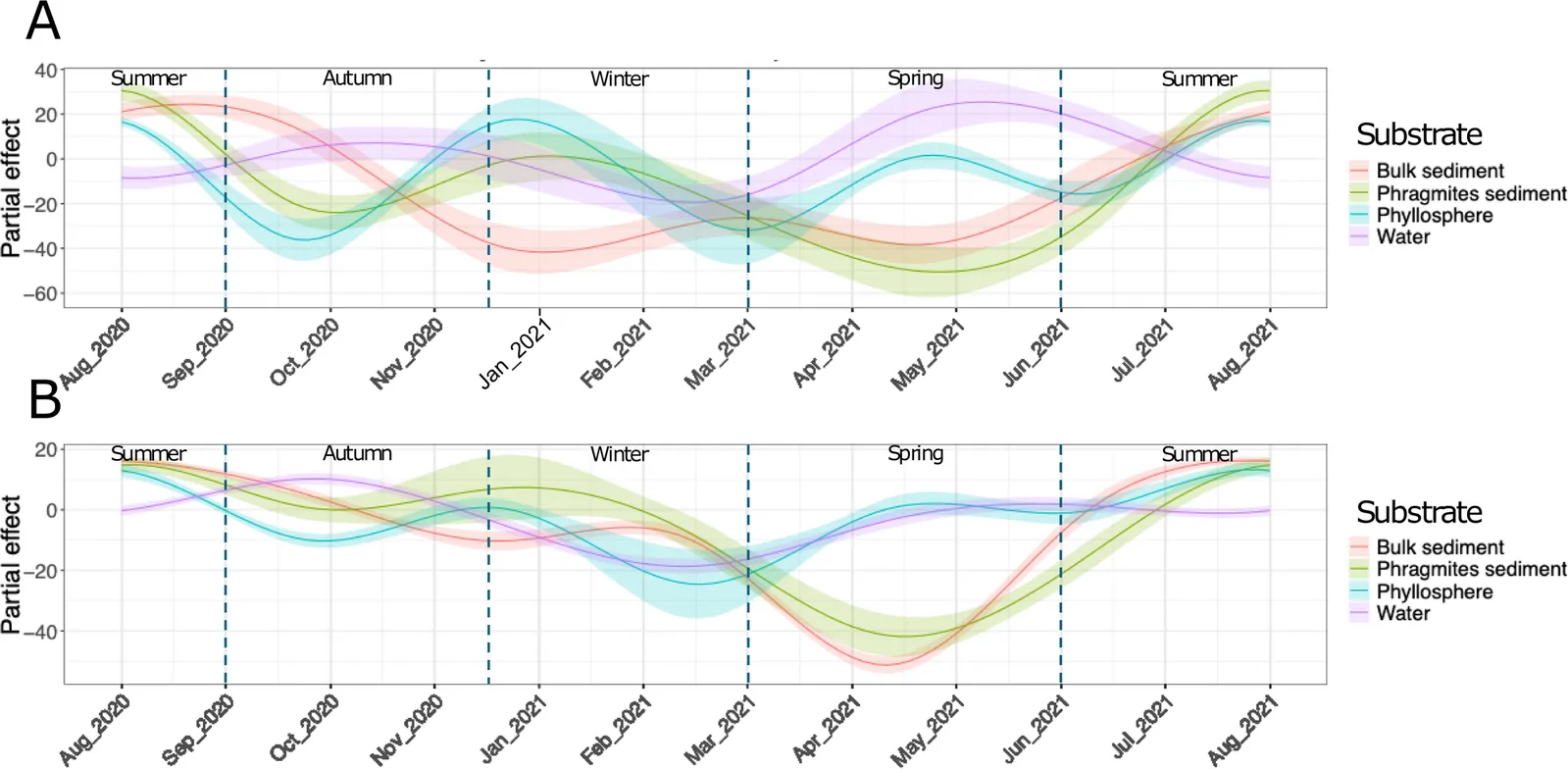
Seasonal patterns of β-diversity for each substrate in Haapsalu and Pärnu Bays, represented by smooths, and their associated 95% confidence intervals (shadow area) calculated by GAMs. Smooth terms are centered at zero; positive partial effects indicate a greater community dissimilarity, whereas negative values indicate a greater similarity. Significant differences between paired temporal smooths were identified from difference smooths when their 95% confidence intervals excluded zero

Results indicated significant seasonal turnover of β-diversity in all substrates (p-value < 0.001) in Haapsalu Bay (adj R^2^ = 0.55; deviance explained = 60%) and Pärnu Bay (adj R^2^ = 0.81; deviance explained = 82%) (Fig. 5). In Haapsalu, differences in seasonal turnover between bulk sediment and phragmites sediment were observed in autumn (higher distance) and winter (lower distance) (Fig. 5; Supplementary Fig. 13). In contrast, Pärnu Bay exhibited minor turnover, only in summer, when the β-diversity of bulk sediment and phragmites sediment showed a higher distance, with bulk sediment being higher than that of phragmites sediment (Fig. 5; Supplementary Fig. 13). Seasonal turnover between water and the phyllosphere was evident in summer and autumn in Haapsalu Bay and Pärnu Bay (Fig. 5; Supplementary Figs. 13 and 14). Overall, seasonal turnover in β-diversity between bulk and phragmites sediments was observed earlier in Pärnu Bay (summer) compared to Haapsalu Bay (in autumn and winter), while water and phyllosphere followed the same turnover between Haapsalu and Pärnu Bays (Fig. 5; Supplementary Figs. 13 and 14).

### Substrate and site effects on β-diversity

Significant differences of β-diversity were found in all substrate pairs in each bay (p-values < 0.001), except between bulk and phragmites sediments in Haapsalu and Pärnu Bays (p-value = 0.79; p-value = 0.07) (Fig. 5; Table 3). Salinity had a significant effect on the β-diversity seasonality between substrates in Pärnu Bay (p-values = 0.001) but not in Haapsalu Bay (p-values = 0.11).

**Table 3:** GAM results of fixed effect factor (substrates) on β-diversity within each site (Haapsalu and Pärnu)

Site	Diversity index	Substrate reference	Substrate comparison	Estimate	Std. Error	t value	*p*-value
Haapsalu	β-diversity	Bulk sediment	Phragmites sediment	1.571	4.984	0.315	0.794
Haapsalu	β-diversity	Bulk sediment	Phyllosphere	-14.492	3.921	-3.695	**< 0.001**
Haapsalu	β-diversity	Bulk sediment	Water	-33.129	5.351	-6.190	**< 0.001**
Haapsalu	β-diversity	Phragmites sediment	Phyllosphere	-16.064	4.360	-3.684	**< 0.001**
Haapsalu	β-diversity	Phragmites sediment	Water	-34.701	5.680	-6.108	**< 0.001**
Haapsalu	β-diversity	Phyllosphere	Water	-18.637	4.775	-3.902	**< 0.001**
Pärnu	β-diversity	Bulk sediment	Phragmites sediment	-3.355	2.374	-1.41	0.07
Pärnu	β-diversity	Bulk sediment	Phyllosphere	-24.651	1.718	-14.347	**< 0.001**
Pärnu	β-diversity	Bulk sediment	Water	-34.830	1.282	27.157	**< 0.001**
Pärnu	β-diversity	phragmites sediment	Phyllosphere	-21.296	2.684	-7.933	**< 0.001**
Pärnu	β-diversity	Phragmites sediment	Water	-31.475	2.428	-12.959	**< 0.001**
Pärnu	β-diversity	Phyllosphere	Water	-10.179	1.791	-5.680	**< 0.001**

**Fig. 6:**
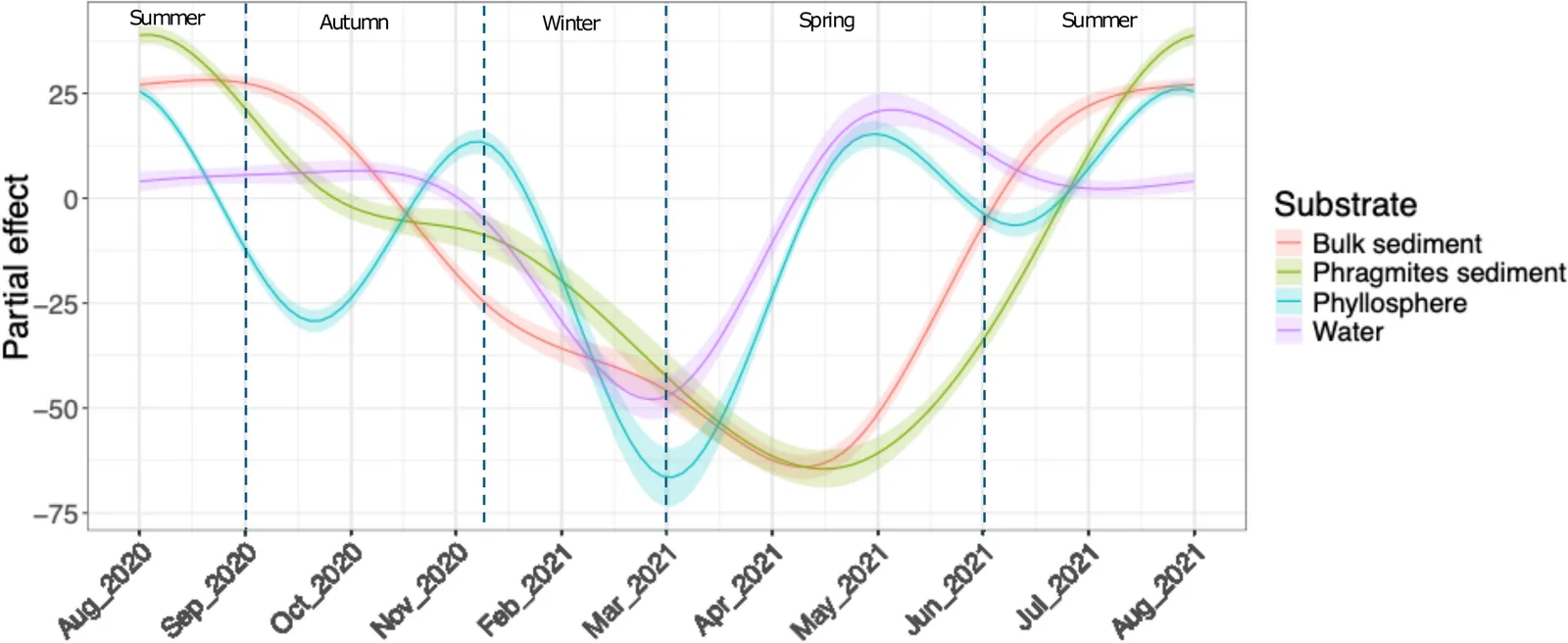
Seasonal patterns of β-diversity of each substrate between Haapsalu and Pärnu Bays are represented by smooths and their associated 95% confidence intervals (shadow area) calculated by GAMs. Smooth terms are centered at zero; positive partial effects indicate a greater community dissimilarity, whereas negative values indicate a greater similarity. Significant differences between paired temporal smooths were identified from difference smooths when their 95% confidence intervals excluded zero

β-diversity of bacterial communities in bulk sediment (p-value < 0.001), phragmites sediment (p-value = 0.02), water (p-value < 0.001), and phyllosphere (p-value < 0.001) differed significantly between Haapsalu and Pärnu Bays (adj R^2^ = 0.83; deviance explained = 83%) (Fig. 6; Table 4), with salinity having a significant effect in Haapsalu Bay (p-value = 0.002).

**Table 4:** GAM results of fixed effect factor (sites) on β-diversity indexes in each substrate between the two sites (Haapsalu and Pärnu)

Diversity index	Site comparison	Substrate	Estimate	Std. Error	t value	*p*-value
β-diversity	Haapsalu-Pärnu	Bulk sediment	122.590	5.47	22.412	**< 0.001**
β-diversity	Haapsalu-Pärnu	Phragmites sediment	-3.47	1.680	-2.070	**0.02**
β-diversity	Haapsalu-Pärnu	Phyllosphere	-31.178	1.573	-19.814	**< 0.001**
β-diversity	Haapsalu-Pärnu	Water	-39.514	1.489	-26.535	**< 0.001**

### Changes in the bacterial community structure: seasonal and reed-associated effects

**Fig. 7:**
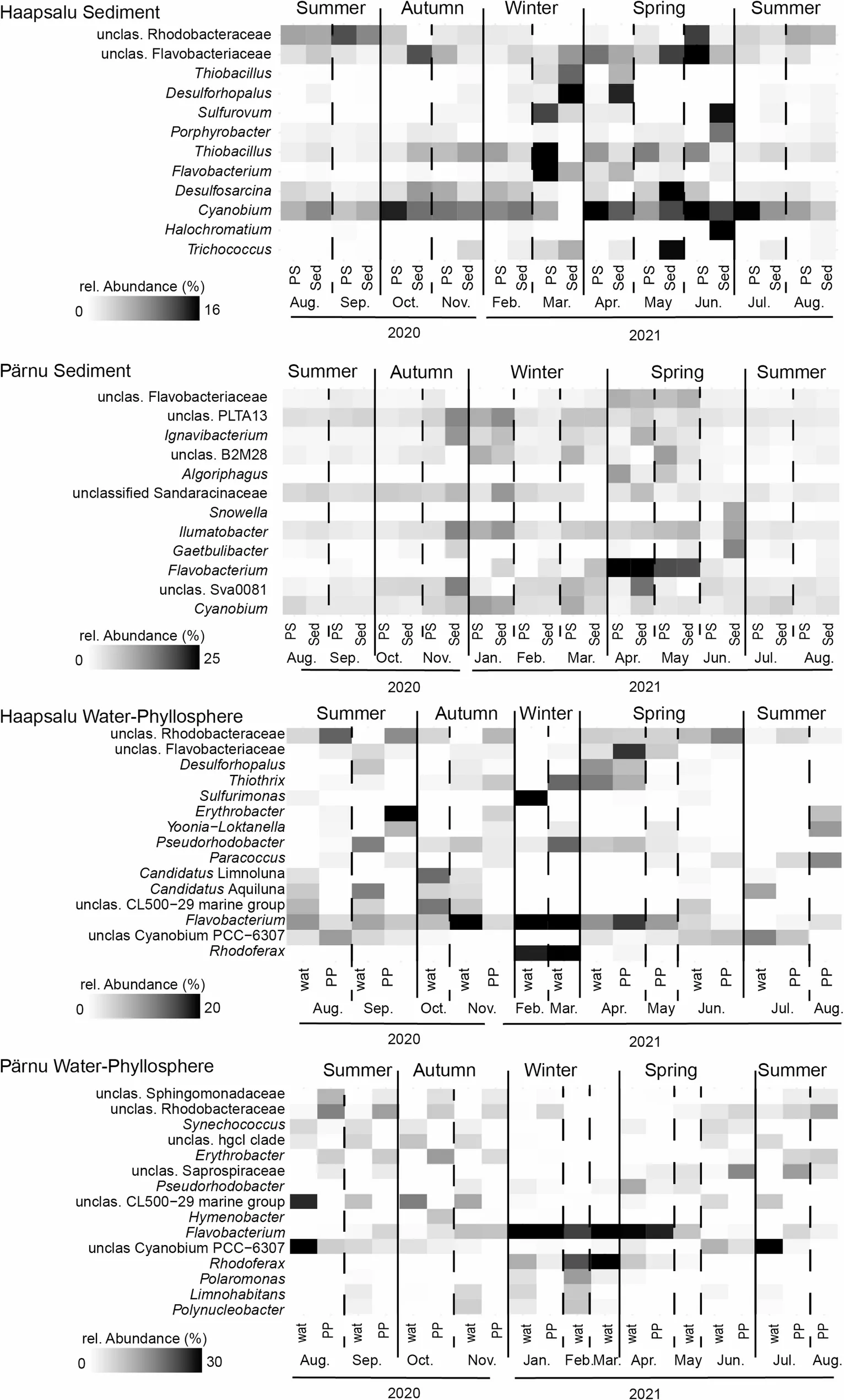
Heatmap comparisons of bacterial taxa relative abundance between bulk sediment (Sed) and phragmites sediment (PS), and between water (wat) and phyllosphere (PP) in Haapsalu and Pärnu Bays

The genera with the highest relative abundance, consistently present across all substrates in both Haapsalu and Pärnu Bays, belonged to Flavobactericeae, which were particularly abundant in winter and spring (Fig. 7). Comparisons of taxa across substrates revealed higher similarity between bulk sediment and phragmites sediment than between water and phyllospheres in both bays. *Erythrobacter* occurred exclusively in the phyllosphere substrate in both bays, showing a consistent abundance pattern throughout the months. Rhodobacteraceae were more abundant in the phyllosphere of Pärnu Bay, whereas in Haapsalu Bay, they were also detected in water. *Rhodoferax* was predominantly observed in water, with its highest abundance during winter and little or no occurrence during the other seasons.

Indicator species analysis of the dominant taxa supported habitat-specific associations, although these differed between bays. In Haapsalu, *Trichococcus*, *Desulforhopalus*, and *Thiobacillus* were significant indicators of phragmites sediment (p-value < 0.05), whereas no significant taxa differentiated bulk and phragmites sediments in Pärnu. Differences between water and phyllosphere were more pronounced, particularly in Pärnu. In Haapsalu, *Candidatus Aquiluna* was associated with water (*p* = 0.017), whereas Rhodobacteraceae was associated with the phyllosphere (*p* = 0.021). In Pärnu, CL500-29 marine group, HGC-I, and *Cyanobium* were significant indicators of water, while *Erythrobacter*, Sphingomonadaceae, Rhodobacteraceae, Saprospiraceae, and *Hymenobacter* were associated with the phyllosphere (p-value < 0.05).

Community composition also showed pronounced bay-specific taxonomic patterns, particularly in sediment-associated communities. In bulk sediment, *Thiobacillus* and *Cyanobium* were significant indicators of Haapsalu, whereas the gammaproteobacterial B2M28 was associated with sediment in Pärnu. Site differentiation was strongest in phragmites sediment, where six dominant taxa (*Trichococcus*, *Sulfurovum*, *Desulforhopalus*, *Porphyrobacter*, *Thiobacillus*, and *Desulfosarcina*) were associated with Haapsalu, while PLTA13, *Ignavibacterium*, *Gaetbulibacter*, and *Ilumatobacter* were associated with Pärnu. In comparison, fewer taxa indicators were detected in the water and phyllosphere: *Thiothrix* and *Candidatus Aquiluna* were associated with Haapsalu water, whereas Saprospiraceae was associated with the Pärnu phyllosphere. *Cyanobium* was common and relatively stable across months in both bulk and phragmites sediments and was also detected in water and the phyllosphere, although at lower abundance in the Pärnu phyllosphere (Fig. 7). PLTA13 and *Ilumatobacter* were prominent in Pärnu sediments, particularly during winter and spring; the indicator analysis further identified both taxa as characteristic of Pärnu phragmites sediment relative to Haapsalu. Water communities showed pronounced seasonal changes. From June to November, CL500-29, *Cyanobium*, *Synechococcus*, HGC-I, and SAR11-III were abundant, whereas *Flavobacterium* and *Rhodoferax* increased during winter and early spring.

Environmental fitting (envfit) of the dominant taxa ordinations further indicated that associations between community composition and environmental conditions differed between bays and habitats. In sediment communities, salinity was significantly associated with compositional variation in Haapsalu (R² = 0.31, p-value = 0.029), whereas temperature (R² = 0.51, p-value < 0.001), salinity (R² = 0.48, p-value = 0.001), and dissolved oxygen (R² = 0.42, p-value = 0.004) were significant in Pärnu. For water and phyllosphere communities, temperature (R² = 0.63, p-value = 0.016) and pH (R² = 0.56, p-value = 0.028) were significantly associated with community variation in Haapsalu, whereas dissolved oxygen (R² = 0.59, p-value = 0.004) and temperature (R² = 0.50, p-value = 0.013) were significant in Pärnu.

**Fig. 8:**
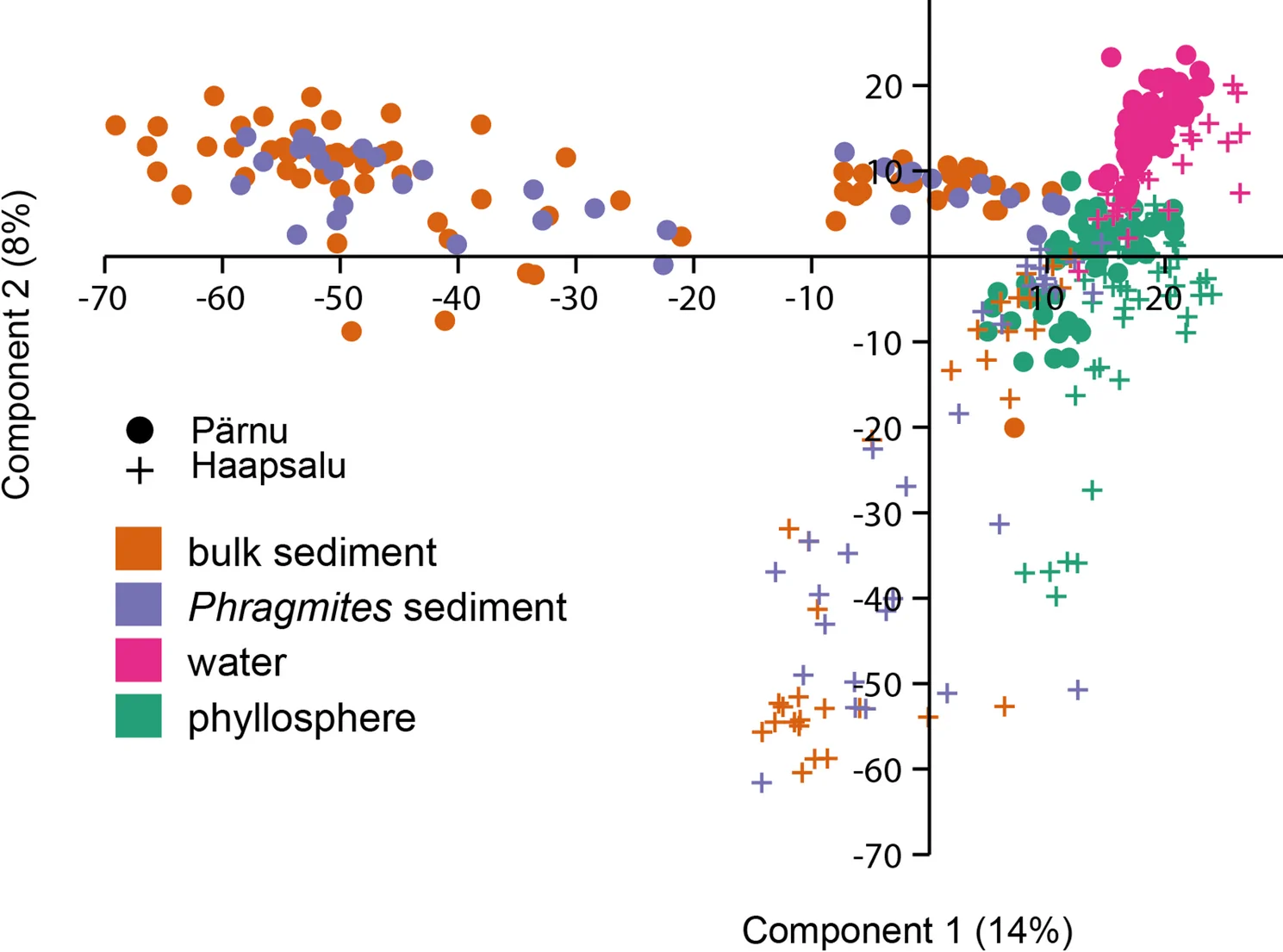
Principal component analysis of bacterial community composition from different substrates and timepoints

Principal component analysis (PCA) of all samples indicated that the bacterial communities from bulk and phragmites sediments, phyllosphere, and water form separate groups (Fig. 8) and are significantly different (PERMANOVA, F = 20.71; R^2^ = 14%; p-value = 0.01). While the phyllosphere and the water from the different bays cluster together, phragmites sediment and bulk sediment clearly differ between the two bays (Fig. 8). A PCA comparing the bacterial community composition of the phyllosphere and water separately indicated a clear separation of the bacterial communities taken at Haapsalu and Pärnu Bays. However, this was only displayed on the second coordinate.

## Discussion

Coastal transition zones are dynamic interfaces connecting terrestrial and aquatic environments, characterized by steep physicochemical gradients and high ecological functionality. However, their pronounced spatial and temporal variability poses significant challenges for ecological monitoring. In this study, we investigated how the structure and diversity of two geographically proximate shallow bays, Pärnu and Haapsalu Bays, respond to seasonal changes in sediment and water, with a focus on the influence of the presence of *Phragmites australis.*

Contrary to our first hypothesis, the presence of *Phragmites australis* had only a minor effect on the overall structure and diversity of sediment and water bacterial communities. However, because this study was restricted to a single annual cycle and focused solely on top-layer sediment, potential effects in deeper sediment, where root-driven oxygenation and carbon exudation occur, may be missed.

Instead, significant differences in the bacterial community composition emerged between the two bays, despite their proximity and similar seasonal trends in temperature, dissolved oxygen (DO), pH, and salinity (Fig. 2). Pärnu Bay consistently exhibited lower average temperature and salinity, but higher DO and pH compared to Haapsalu Bay. These differences are likely driven by contrasting bay morphology and hydrology.

### Seasonal dynamics of α-diversity and site-specific effect

Bacterial 𝛼-diversity exhibited pronounced substrate-specific seasonal dynamics in both bays. Bulk and phragmites sediment bacterial communities showed broadly similar seasonal patterns, characterized by lower diversity in spring and higher diversity in summer–autumn, suggesting that the presence of *P. australis* does not fundamentally alter the seasonal dynamics of sediment-associated microbial communities (Fig. 3).This aligns with findings from seagrass systems reported by Chen et al. [10], who showed that seasonality had a stronger influence on bacterial communities in seagrass bed sediments than the vegetation cover. However, subtle differences in seasonal patterns were observed between bulk and phragmites sediments in our study, particularly during summer-autumn in Haapsalu Bay and spring-summer in Pärnu Bay.

Notably, the seasonal variation of 𝛼-diversity in sediments and water was generally stronger in Pärnu Bay than in Haapsalu Bay. This disparity may be driven in part by the influence of the Pärnu River and the associated fluctuations in organic matter inputs. Pärnu River total organic carbon (TOC) concentrations varied seasonally from 12 to 23 mgC/L, indicating temporal variability in the organic carbon supplied to the bay. This delivery of organic matter may accelerate microbial responses, as shown in the river-influenced estuaries [22]. In contrast, Haapsalu Bay exhibited higher seasonal turnover in ß-diversity within water and phyllosphere communities. This turnover was potentially linked to a marked spring drop in DO, which may have triggered community shifts. Seasonal changes in water quality parameters, including DO and nutrients, have been shown to strongly influence bacterial community composition and diversity in aquatic systems [23, 42], as well as in phyllosphere microbial assemblages that also display seasonal variation in structure and diversity [46]. While sediment-associated habitats displayed pronounced seasonal 𝛼-diversity variations consistent with known temporal dynamics in sediment bacteria [25], water communities showed weak or non- seasonal variation in 𝛼-diversity, reaching significance only in Pärnu Bay.

However, 𝛼-diversity differed strongly among substrates in both bays, with sediments supporting higher diversity than phyllosphere and water communities, consistent with previous studies establishing habitat type as primary determinant of microbial diversity [12, 49, 51]. Also, in line with previous studies [6, 9, 19], bulk sediment possessed significantly higher 𝛼-diversity than phragmites sediment. In contrast to the sediment, no significant differences were found between water and phyllosphere communities based on the Chao1 index, although a weak substrate effect was observed for the Shannon index in Pärnu Bay. These results are consistent with Iqbal et al. [24], who observed high compositional similarity between water and phyllosphere bacterial communities. This similarity is likely driven by strong dispersal connectivity and continuous microbial exchange between the water column and plant surfaces. Overall, both substrate and seasonal effects were more pronounced in Pärnu Bay, suggesting that while *P. australis* does not broadly reshape microbial community structure, it can modulate bacterial richness under specific environmental conditions.

### Seasonal dynamics of β-diversity and site-specific turnover

Similarly, to the 𝛼-diversity seasonality patterns, bulk sediment and phragmites sediment exhibited the lowest β-diversity turnover in winter-spring and the highest during summer-early autumn in both bays (Fig. 5). In contrast, water and phyllosphere communities showed higher turnover in spring and lower in winter-spring, indicating divergent seasonal dynamics. The strongest divergence between water and phyllosphere communities occurred in winter in Haapsalu Bay and during spring-summer in Pärnu Bay, highlighting habitat-specific responses to seasonal drivers. This divergence was more pronounced in Haapsalu Bay, likely due to stronger environmental fluctuations. However, the absence of sampling during the winter months in both bays (due to ice cover) may have led to an overestimation of seasonal effects in the phyllosphere models.

PCA and GAM results confirmed that sediment bacterial communities were most distinct between bays, with minimal overlap in dominant ASVs, suggesting that sediment communities are highly adapted to local environmental conditions and act as a buffer against environmental variability. This is consistent with findings from the Baltic Sea, where sediment microbial communities show strong site-specificity and low turnover across seasons [47, 49]. In contrast, water and phyllosphere communities showed greater similarity between bays (Fig. 8), likely due to higher connectivity for water and specific environmental selection for the phyllosphere.

### Drivers of community assembly: environmental vs. phyllosphere effects

Our findings support the hypothesis that long-term abiotic environmental drivers, particularly temperature, salinity, and riverine inputs, are primary regulators of β-diversity microbial community dynamics, especially in sediments. The sediment microbiome’s relative stability across seasons and sites underscores its role as a resilient reservoir that buffers against short-term fluctuations, as previously suggested [47, 49].

In contrast, water-associated communities were more responsive to dynamic environmental conditions, including seasonal temperature shifts, changes in DO, and organic matter inputs.

The presence of different indicator taxa between bays supports that geographically close bays harbor different bacterial communities. This is also indicated in the dominance of *Cyanobium* in Haapsalu Bay and *Synechococcus* in Pärnu Bay. The differences were likely driven by bay-specific hydrology and nutrient availability since phytoplankton composition is strongly influenced by nutrient loading and water residence time [38, 48]. The observed dominance of *Synechococcus* in Pärnu Bay may be linked to the more consistent nutrient supply from river inflow [28].

The phyllosphere, while influenced by the host plant, showed strong seasonal dynamics and high turnover of the bacterial community, likely shaped by plant phenology, exudate release, and exposure to the water column. However, the absence of phyllosphere samples during winter limits our ability to fully capture seasonal patterns, but the observed spring increase in diversity likely reflects the onset of *P. australis* vegetation and associated microbial colonization. This aligns with studies on marine macrophytes, which show that phyllosphere microbial communities are linked to the macrophyte growth cycle [29].

### The role of *Phragmites australis*: a context-dependent influence

While previous studies have documented the influence of macrophytes on sediment microbial communities (e.g., in seagrass meadows, [21]), our results suggest a minor role for *P. australis*. In Haapsalu Bay, no significant differences were observed between bulk and phragmites sediments in either 𝛼- or β-diversity. However, in Pärnu Bay, a marginal but significant effect on 𝛼-diversity was detected, possibly due to the interplay between riverine inputs and plant-mediated processes.

This context-dependent effect highlights that macrophyte influence is not universal but depends on local environmental conditions. In Pärnu Bay, the combination of high nutrient loading and seasonal hydrological variability may amplify plant-microbe interactions, whereas in the Haapsalu Bay, such effects are masked by stronger abiotic drivers. This is consistent with findings from a study on *P. australis* in a shallow coastal lake, where plant presence was linked to higher bacterial 𝛼-diversity and lower β-diversity in sediment communities [14].

## Conclusion

Our study demonstrates that geographically close bays can harbor distinct bacterial communities, shaped more by local hydrology, environmental gradients, and riverine inputs than by the presence of *P. australis*. Sediment communities exhibit greater stability and site-specificity, acting as a buffer against environmental change, whereas water and phyllosphere communities are more responsive to seasonal dynamics. These findings underscore the importance of site-specific assessments in coastal microbial ecology and caution against generalizing results across bays, even those in close proximity. Given the projected intensification of freshwater and allochthonous organic matter inputs and salinity fluctuations in the Baltic Sea coastal areas under climate change, sampling over several annual periods is important to determine whether the observed site-specific patterns remain stable under altered hydrological and nutrient load regimes. Moreover, more studies investigating bacterial community succession in shallow coastal bays are necessary to support this result and detect possible patterns.

## Supplementary Information

Below is the link to the electronic supplementary material.

Supplementary Material 1

## Data Availability

Data is available under accession number PRJEB109789 at the European Nucleotide Archive at the following URL https://www.ebi.ac.uk/ena/browser/view/PRJEB109789.
