# Supplementary material for: Local hydrology and seasonality outweigh the influence of Phragmites australis on coastal bacterial communities across two Baltic Sea bays
Source: Environ Microbiome. 2026 Nov 2;21:129. doi: 10.1186/s40793-026-00979-4 (PMC13729460; doi:10.1186/s40793-026-00979-4)
Supplement: Supplementary file 1 [file 40793_2026_979_MOESM1_ESM.docx]

**Local hydrology and seasonality outweigh the influence of *Phragmites australis* on coastal bacterial communities across two Baltic Sea bays**

Fabio Ercoli^1,2^, Helen Tammert^1,3^, Veljo Kisand^1,3^, Kätlin Blank^1^, Carmen Kivistik^1^, Daniel P.R. Herlemann^1,4^*

*corresponding author

Fabio Ercoli ORCID and email: [0000-0003-2433-6222](https://orcid.org/0000-0003-2433-6222) fabio.ercoli@emu.ee

Helen Tammert ORCID and email: 0000-0001-5933-7079 helen.tammert@emu.ee

Veljio Kisand ORCID and email: 0000-0002-5535-1639 veljo.kisand@ut.ee

Kätlin Blank ORCID and email: 0000-0002-8084-5139 katlin.blank@emu.ee

Carmen Kivistik email: kivistikcarmen@gmail.com

Daniel Herlemann ORCID and email: 0000-0002-8100-4649 daniel.herlemann@io-warnemuende.de

^1^ Chair of Hydrobiology and Fisheries, Institute of Agricultural and Environmental Sciences, Estonian University of Life Sciences, Kreutzwaldi 5D, 51006, Tartu, Estonia

^2^ Department of Biological and Environmental Science, University of Jyväskylä, P.O. Box 35, 40014, Jyväskylä, Finland

^3^ Institute of Technology, University of Tartu, Tartu, Estonia

^4^ Leibniz-Institute for Baltic Sea Research Seestrasse 15 D-18119 Rostock Germany

**Supplementary Figure 1**. Rarefaction curve of the samples. The dashed line indicates the cutoff of 950 reads used for rarefying the data.

**Supplementary Figure 2.** Seasonal variation of environmental variables in Haapsalu and Pärnu bays represented by smooth lines with a 95% conﬁdence interval represented by grey shadow.

**Supplementary Figure 3**. Boxplot representing Chao1 diversity index in different substrates from August 2020 to August 2021.

**Supplementary Figure 4.** Boxplot representing Shannon diversity index in different substrates from August 2020 to September 2021.

**Supplementary Figure 5.** Shannon seasonality in Haapsalu (A) and Pärnu (B) Bays per each substrate represented by smooth lines and their associated 95% confidence intervals (shadow area) calculated by GAMs. Smooth terms are centred at zero, and positive and negative values indicate higher and lower α-diversity.

**Supplement Figure 6.** Smooth differences of Chao1 index from paired bulk sediment and phragmites sediment, and phyllosphere and water substrates comparisons in Haapsalu Bay and their associated 95% confidence intervals (grey shadow) calculated by GAMs. Significant differences between paired temporal smooths were identified from difference smooths when their 95% confidence interval exclude zero.

**Supplement Figure 7.** Smooth differences of Shannon index from paired bulk sediment and phragmites sediment, and phyllosphere and water substrates comparisons in Haapsalu Bay and their associated 95% confidence intervals (grey shadow) calculated by GAMs. Significant differences between paired temporal smooths were identified from difference smooths when their 95% confidence interval exclude zero.

**Supplement Figure 8.** Smooth differences of Chao1 index from paired bulk sediment and phragmites sediment, and phyllosphere and water substrates comparisons in Pärnu Bay and their associated 95% confidence intervals (grey shadow) calculated by GAMs. Significant differences between paired temporal smooths were identified from difference smooths when their 95% confidence interval exclude zero.

**Supplement Figure 9.** Smooth differences of Shannon index from paired bulk sediment and phragmites sediment, and phyllosphere and water substrates comparisons in Pärnu Bay and their associated 95% confidence intervals (grey shadow) calculated by GAMs. Significant differences between paired temporal smooths were identified from difference smooths when their 95% confidence interval exclude zero.

**Supplementary Figure 10**. Shannon index seasonality in each substrate in Haapsalu and Pärnu Bays is represented by smooths and their associated 95% confidence intervals (shadow area) calculated by GAMs. Significant differences between paired temporal smooths were identified from difference smooths when their 95% confidence interval exclude zero.

**Supplement Figure 11.** The smooth differences of Chao1 from paired comparisons between Haapsalu and Pärnu Bays of each substrate and their associated confidence intervals (grey shadow) calculated by GAMs. Significant differences between paired temporal smooths were identified from difference smooths when their 95% confidence interval exclude zero.

**Supplement Figure 12**. The smooth differences of Shannon from paired comparisons between Haapsalu and Pärnu Bays of each substrate and their associated confidence intervals (grey shadow) calculated by GAMs. Significant differences between paired temporal smooths were identified from difference smooths when their 95% confidence interval exclude zero.

**Supplement Figure 13.** The smooth differences of β-diversity from from paired bulk sediment and phragmites sediment, and phyllosphere and water substrates comparisons in Haapsalu Bay and their associated confidence intervals (grey shadow) calculated by GAMs. Significant differences between paired temporal smooths were identified from difference smooths when their 95% confidence interval exclude zero.

**Supplement Figure 14.** The smooth differences of β-diversity from from paired bulk sediment and phragmites sediment, and phyllosphere and water substrates comparisons in Pärnu Bay and their associated confidence intervals (grey shadow) calculated by GAMs. Significant differences between paired temporal smooths were identified from difference smooths when their 95% confidence interval exclude zero.
